# Policy Impediments to Tuberculosis Elimination: Consequences of an Absent Medicare National Coverage Determination for Tuberculosis Prevention

**DOI:** 10.1007/s10903-025-01674-1

**Published:** 2025-03-05

**Authors:** Matthew T. Murrill, Katya Salcedo, Cynthia A. Tschampl, Nisha Ahamed, Elinor S. Coates, Jennifer Flood, Donna H. Wegener, Priya B. Shete

**Affiliations:** 1 Division of Hospital Medicine, University of California San Francisco, San Francisco, USA; 2 Center for Tuberculosis, University of California San Francisco, San Francisco, USA; 3 Institute for Global Health Sciences, University of California San Francisco, San Francisco, USA; 4 Tuberculosis Control Branch, California Department of Public Health, Richmond, USA; 5 Schneider Institutes for Health Policy and Research, The Heller School for Social Policy and Management, Brandeis University, Waltham, USA; 6 Stop TB USA, Atlanta, USA; 7 Global Tuberculosis Institute, Rutgers New Jersey Medical School, Newark, USA; 8 National Tuberculosis Coalition of America, Atlanta, USA; 9 Division of Pulmonary, Critical Care, Allergy and Sleep Medicine, University of California San Francisco, San Francisco, USA

**Keywords:** Medicare, Tuberculosis, TB, Screening, Prevention, National coverage determination

## Abstract

Despite being preventable, tuberculosis (TB) continues to affect thousands of individuals in the US with an increasing incidence every year since the COVID-19 pandemic. Critical to TB elimination efforts is the scale-up of screening and treatment for the estimated 13 million Americans with TB infection. For the Medicare population, the implementation of TB preventive services is hampered by the lack of clear coverage of TB infection screening. Despite a consistent US Preventive Services Task Force (USPSTF) recommendation since 1996, TB infection screening does not have a Medicare national coverage determination, which would ensure consistent coverage across the US. All other USPSTF recommended preventive services for Medicare recipients have a national coverage determination, a broadly applicable clinical quality measure or are medications generally covered through Medicare Part D. The lack of a national coverage determination for TB infection screening is crucially a health equity issue with TB disproportionally affecting non-US-born and minoritized US-born persons. To address this important barrier to TB prevention, a request to Medicare for a national coverage determination for TB screening was submitted in early 2024.

Despite being preventable, tuberculosis (TB) is again the world’s leading infectious cause of death (1.25 million persons in 2023) after being briefly surpassed by COVID-19 during the pandemic [1, 2]. If undiagnosed and untreated, individuals with TB infection can progress to develop TB disease [3]. The risk of progression from TB infection to TB disease is significantly higher for those with a weakened immune system due to comorbidities (e.g., HIV infection, end-stage renal disease or diabetes), immunosuppressive medications or older age [4].

In the US, TB is an important cause of morbidity and mortality with approximately 8000 to 9000 individuals diagnosed with TB disease annually. Concerningly, TB disease incidence has risen every year since the start of the COVID-19 pandemic in 2020 with a 16% rise in 2023 alone [5]. This increase reflects several health system challenges during this time: the reallocation of public health resources to pandemic response, reduced TB preventive care, delayed diagnoses for TB disease and reduced healthcare utilization and access. More than 80% of TB disease in the US is due to the reactivation of TB infection with only 20% due to the recent transmission of TB from person-to-person [5, 6]. Once an individual develops TB disease, the outcomes are often grim with more than half of individuals diagnosed being hospitalized [7, 8] and one in ten dying of the disease [8]. TB disease also has substantial financial costs to individuals, communities, and health systems. Treatment for the thousands who develop TB disease costs an estimated 16,000 to 23,000 US dollars (USD) per person not including out-of-pocket costs [8, 9], indirect costs such as time lost from work [10], the consequences of social isolation and stigma as well as the public health costs of contact investigation.

TB prevention through the testing and treatment of the more than 13 million Americans who are estimated to have TB infection [11] could prevent significant suffering and save millions of dollars over time. The screening of individuals with increased risk of TB infection or progression to TB disease was first recommended by the United States Preventive Services Task Force (USPSTF) in 1996 [12], and again in 2016 [13], and 2023 [14], each time with the highest recommendation for implementation (i.e., grade A or B recommendation). The USPSTF is joined by the Centers for Disease Control and Prevention (CDC), the Infectious Diseases Society of America (IDSA), the National Tuberculosis Coalition of America (NTCA), and the American Thoracic Society (ATS) [15, 16] in recommending screening for TB infection as an essential preventive care tool for reducing the burden of TB disease in the United States. This goal is also in keeping with US national public health strategic priorities [17] and is rooted in a clear and growing body of evidence that TB prevention saves lives and averts costs. This evidence demonstrates that the prioritized testing and treatment of individuals at increased risk for TB infection is the single most important driver towards achieving US TB elimination goals [18–21] and is not only cost-effective but potentially cost-saving [18, 20–21]. Unfortunately, despite the availability of more specific interferon-gamma release assay tests (IGRA) and generally better tolerated rifamycin-based treatment regimens [16], there remain significant gaps in TB preventive care in the US [22–26].

Specifically for the Medicare population, one key health system barrier impeding scale up is the lack of clear Medicare coverage for TB infection screening. In 2010, the Patient Protection and Affordable Care Act (ACA) mandated that all non-grandfathered private health insurance and Medicaid expansion plans cover USPSTF grade A and B recommended services without patient cost-sharing [27]. Under the ACA, Medicare may cover USPSTF grade A and B recommended preventive services at a national level if these services are deemed both reasonable and necessary for Medicare recipients through a process called a national coverage determination (NCD) [28, 29]. Without an NCD, a Medicare Administrative Contractor, a private insurer who has been awarded a contract to process claims for a specific geographic region, may also make local coverage determinations (LCDs) that provide clear coverage for certain services in their regional jurisdiction. Despite TB infection screening having a consistent USPSTF grade A or B recommendation for nearly three decades, it has yet to be reviewed by CMS for an NCD or even regionally for an LCD [29, 30]. Without an NCD or LCD, coverage determinations are made by individual Medicare insurance plans resulting in variable coverage and financial burden to patients.

For the 26 USPSTF grade A or B recommended services applicable to the Medicare population ≥ 65 years of age, we evaluated the presence of Medicare coverage in the Medicare Coverage Database [29] as well as the presence of routinely used clinical quality measures in the Centers for Medicare and Medicaid Services’ (CMS) Merit-based Incentive Payment System (MIPS) [31] and the Healthcare Effectiveness Data and Information Set (HEDIS) [32]. Out of the applicable USPSTF grade A and B services, only 4 of 26 (15%) do not have an existing Medicare NCD (Table 1) [29, 30] with an NCD for the prevention of HIV with pre-exposure prophylaxis recently finalized in late 2024. Two of these four recommendations are for medications that are often covered by Medicare Part D, making an NCD determination likely less essential. Furthermore, a MIPS or HEDIS quality measure, which may incentivize providers by linking a service with reimbursement, exists for the only other USPSTF grade A or B recommendation without an NCD that is not a medication (falls prevention in older adults).

**Table 1:** Summary of Four US Preventive Service Task Force Grade A or B recommendations without a Medicare national coverage determination [29–32]

USPSTF Recommendation	History of Grade of USPSTF Recommendation [30]	Medication	Existing Quality Measures [31, 32]
Latent Tuberculosis Infection in Adults: Screening	1996: A 2016: B 2022: B	No	Medicare quality measure: TB infection screening only prior to starting immunosuppressive medications (MIPS #176)
Falls Prevention in Community-Dwelling Older Adults: Exercise Interventions	2012: B 2018: B 2024: B	No	Medicare quality measure: create plans of care for falls (MIPS #155) HEDIS measure: Fall Risk Management (FRM)
Breast Cancer: Medication Use to Reduce Risk	2002: B 2013: B 2019: B	Yes*	--
Statin Use for the Primary Prevention of Cardiovascular Disease in Adults: Preventive Medication	2016: B^ 2022: B	Yes*	HEDIS measure: Statin Therapy for Patients with Cardiovascular Disease (SPC)

*Footnotes*:

- MIPS (Merit-based Incentive Payment System): component of Medicare’s Quality Payment Program that links medical provider reimbursement to quality of care through quality measures, cost, improvement activities and interoperability of healthcare data

- HEDIS (Healthcare Effectiveness Data and Information Set): widely used set of standardized measures to evaluate health system performance, developed by the National Committee for Quality Assurance, a private non-profit organization

*Medications: often covered by Medicare Part D, although subject to cost-sharing and deductibles

^Statin use: prior recommendations in 1996, 2001 and 2008, but specific recommendations significantly evolved based on the development and use of different tools for risk stratification for cardiovascular disease

**Sources / notes**: authors’ analysis of US Preventive Service Task Force grade A and B recommendations, existing Medicare national coverage determinations and clinical quality measures (Merit-based Incentive Payment System or Healthcare Effectiveness Data and Information Set)

The lack of a national coverage determination for TB infection screening is also crucially a health equity issue. TB disproportionally affects non-US-born persons and minoritized US-born persons with significant health and financial consequences [33–35]. TB disease incidence is eight times higher for Black Americans than White Americans, and the incidence for non-US-born Asian persons is seventy times higher than US-born persons [5]. Approximately 90% of all TB diagnoses occur among racial or ethnic minority populations, and approximately 70% occur among individuals who were born outside the US [6, 36]. Addressing TB in the US from a health equity framework is an essential element of the CDC’s Goals for Health Equity in Tuberculosis Prevention and Control [36] and is in keeping with the Federal government’s Executive Order on Advancing Racial Equity and Support for Underserved Communities [37]. Because of the intersecting social and structural determinants of health that drive TB disease, the communities most affected by TB often rely on Medicare or Medicaid to facilitate access to basic preventive health services. The lack of a CMS determination for a disease that affects these communities can create additional barriers to quality preventive care, further perpetuating health inequities.

Specifically for non-US born persons, Medicare eligibility can be challengingly variable and based on immigration status, duration of residence and employment history. While naturalized citizens, lawful permanent residents (i.e., green card holders) and individuals with “qualified alien” status (e.g., asylees, refugees) are all eligible for Medicare enrollment after five years of consecutive residence in the US, lawful temporary (e.g., temporary visa holders) or unauthorized residents are not eligible for Medicare [38]. Despite this five-year residence requirement, data from the US Census Bureau’s 2023 American Community Survey indicate that 90% of non-US born persons ≥ 65 years of age (7.7 million persons) reported current Medicare coverage, accounting for approximately 14% of all Medicare recipients ≥ 65 years of age [39]. Even though a Medicare national coverage determination would not impact legal temporary residents, unauthorized residents or lawful permanent residents not enrolled in Medicare, it has the potential to significantly reduce barriers to TB screening for the majority of non-US born persons ≥ 65 years of age with Medicare coverage.

A national coverage determination for TB infection screening would furthermore benefit many Medicare recipients who often have multiple risk factors for TB reactivation or poor TB outcomes. Persons ≥ 65 years of age in the US, who are eligible for Medicare, account for over 25% of TB diagnoses each year and suffer an increased risk of death. Analysis of the CDC’s most recent National Health and Nutrition Examination Survey (NHANES) that included data on TB infection identified that non-US-born persons receiving Medicare had the highest prevalence of TB infection (29%) compared to individuals on other insurance plans (12–20%) [40]. Many Medicare recipients also have key comorbidities that increase the risk of TB reactivation [41], including: HIV infection [42], end-stage renal disease [43], diabetes [44], lupus [45] and cancer treated with immunotherapies [46]. Medicare furthermore bears a disproportionate share of the costs of TB treatment compared to private insurers. In 2014, TB-related hospitalizations alone cost Medicare an estimated 79 million USD (compared to 43.6 million USD for private insurers), and Medicare and Medicaid together bore 69% of all TB hospitalization costs [9]. Recent modeling analyses of the Medicare population have highlighted that TB infection screening for non-US persons is more cost-effective compared to long-term care residents, incarcerated persons and persons experiencing homelessness [47].

To address this key barrier in TB prevention for the Medicare population, a coalition of 25 public health, academic, clinical and advocacy organizations have submitted a formal request to CMS to make an NCD in favor of TB infection screening using IGRAs for Medicare recipients with an increased risk of infection. We strongly encourage CMS to make a timely national coverage determination to include the screening of TB infection in accordance with USPSTF recommendations. A CMS coverage determination would facilitate improved reimbursement, reduced patient cost-sharing, and streamlined billing for risk-based TB infection screening. Reimbursement for these services through Medicare would also enable better monitoring, accountability and quality standards for TB preventive care services. Perhaps most importantly, an NCD for TB infection screening would recognize that TB is a disease that disproportionately affects historically minoritized and marginalized populations and that achieving health equity requires action to address the financial and systemic barriers that impede access to quality TB preventive care for these communities.

## Data Availability

Four publicly available datasets were analyzed to evaluate the presence of Medicare coverage and clinical quality measures for all United States Preventive Services Task Force (USPSTF) grade A or B recommended services relevant to the Medicare population. Published USPSTF recommendations: https://www.uspreventiveservicestaskforce.org/uspstf/topic_search_results?topic_status=P. Medicare national and local coverage determinations (Medicare Coverage Database): https://www.cms.gov/medicare-coverage-database/search.aspx. United States Merit-based Incentive Payment System quality measures: https://qpp.cms.gov/mips/quality-requirements. Healthcare Effectiveness Data and Information Set quality measures: https://www.ncqa.org/hedis/measures/. Additionally, one publicly available dataset was used to estimate the number of non-US born persons ≥65 years of age in the US that are covered by Medicare. 2023 American Community Survey, US Census Bureau. https://data.census.gov/mdat/#/.
